# Augmenting mitral valve repair evaluation with intraoperative left ventricle pressure measurements

**DOI:** 10.1093/icvts/ivac242

**Published:** 2022-09-29

**Authors:** Hugo Issa, Mimi Deng, Kenza Rahmouni, Vincent Chan

**Affiliations:** Division of Cardiac Surgery, University of Ottawa Heart Institute, Ottawa, ON K1Y 4W7, Canada; Division of Cardiac Surgery, University of Ottawa Heart Institute, Ottawa, ON K1Y 4W7, Canada; Division of Cardiac Surgery, University of Ottawa Heart Institute, Ottawa, ON K1Y 4W7, Canada; Division of Cardiac Surgery, University of Ottawa Heart Institute, Ottawa, ON K1Y 4W7, Canada

**Keywords:** Mitral valve, Mitral valve repair

## Abstract

Surgical mitral valve repair remains the gold standard treatment of mitral regurgitation due to degenerative disease. Surgery is performed on the quiescent heart; therefore, assessments of valve repair success can only be made following separation from cardiopulmonary bypass. Intra-ventricular pressure measurements are often made in percutaneous valve procedures but has yet been described at the time of surgical repair. As an example, the saline test, whereby normal saline is injected across the mitral valve from the left atrium into the left ventricle, on the arrested heart remains an integral component of surgical repair. However, the haemodynamics of the saline test have never been evaluated. We present a simple and novel technique to quantify the saline test by passing a 22-G catheter across the mitral leaflets during saline testing under maximal ventricle distension. The saline test may be less informative among patients in whom the maximum generated left ventricle diastolic pressure is low. These data may be of help to a surgeon interpreting intraoperative saline tests with the hope of a competent mitral valve. As well, it may provide support for intraventricular pressure monitoring at the time of mitral valve surgery.

## INTRODUCTION

Surgical mitral valve repair remains the gold standard approach for addressing mitral regurgitation (MR). A variety of techniques have been described to repair the mitral valve and these are associated with favourable results at expert centres [1–5]. However, surgery is typically performed on the quiescent heart; therefore, assessments of valve repair can only be made following separation from cardiopulmonary bypass. Intra-ventricular pressure measurements are often made in percutaneous valve procedures but have yet been described at the time of surgical repair.

In surgical mitral repair procedures, the saline test, whereby normal saline is injected across the mitral valve from the left atrium into the left ventricle (LV), remains ubiquitous as part of the reconstruction [6]. The saline test helps deploy the mitral leaflets and allows the surgeon to gauge areas of prolapse or restriction. Often, the lack of regurgitation back across the mitral valve into the left atrium is thought to be reassuring in many cases [7], but persistent or recurrent MR can be observed once cardiac contractility resumes due the discordant state of heart during surgical manipulation and normal cardiac contraction.

Herein, we describe a novel technique to measure the LV pressure on the arrested heart at the time of surgical mitral repair. This simple technique may provide the team with more information at the time of mitral reconstruction, which may potentially aid the assessment of repair on the arrested heart.

## DESCRIPTION

**Figure 1:**
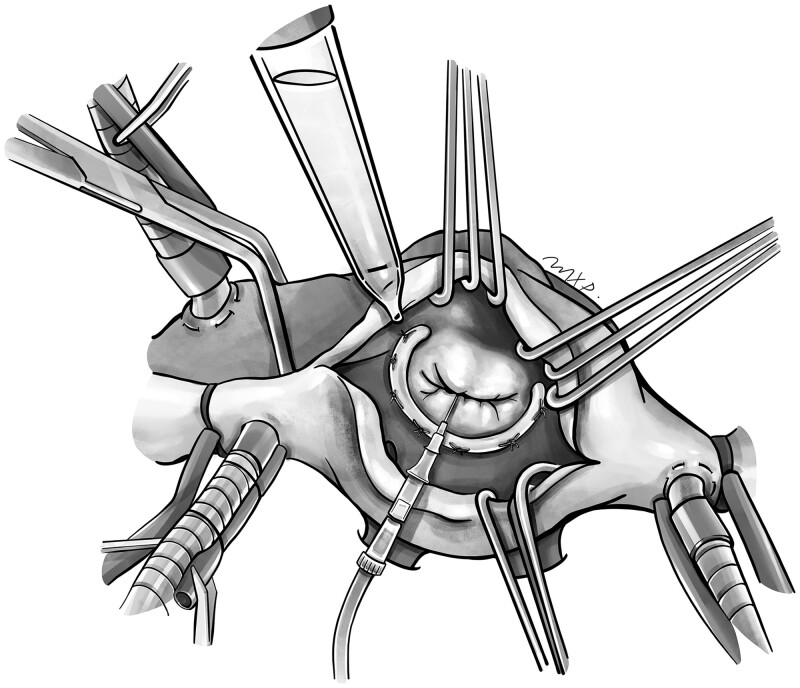
Intraoperative left ventricle pressure measurement at the time of saline testing following surgical mitral valve repair. A 22-G catheter is passed across the mitral leaflets following completion of repair during saline testing under maximum pressurization.

LV pressure measurements were made on patients presenting for mitral valve repair via cardiopulmonary bypass, cardioplegic arrest and left atriotomy. Once the mitral valve repair is complete, a 22-G Jelco intravenous catheter (Minneapolis, MN) is passed across the mitral leaflets attached to a 6-ft pressure monitoring line. The apparatus is stabilized manually with forceps and the system is zeroed (Fig. 1). Saline is continually injected across the mitral leaflets to ensure leaflet deployment until a maximum pressure can be obtained. At that pressure, the mitral valve leaflets are inspected for appropriate closure. The slim diameter of a 22-G intravenous catheter allows the mitral valve leaflets to coapt without deformation.

Between November 2021 and January 2022, 20 consecutive patients who underwent mitral valve repair of degenerative MR were assessed. These patients were of mean age 61.5 ± 10.7 years, and 5 (25%) were female. Patients had an average height and weight of 171 ± 9 cm and 75 ± 11 kg, respectively. The average echocardiographically calculated LV mass was 106 ± 24 g/m^3^. All patients had severe MR prior to surgery with LV diameter measurements in diastole and systole of 54.2 ± 3.3 and 32.4 ± 8.2 mm, respectively. Mitral valve repair was performed with ring annuloplasty using the Medtronic Futureband (Minneapolis, MN) with a median size of 32 (interquartile range 30–34). Neochordae were used in 10 (50%) and leaflet resection performed in 2 (10%). The mean LV pressure obtained with direct monitoring across the mitral leaflets was 27.6 ± 14.6 mmHg. Intraoperative, post-procedure TEE confirmed trace MR in all patients with subsequent TTE performed pre-hospital discharge confirming MR ≤ 1+. Of note, 1 patient required an additional cardiopulmonary bypass run despite an adequate appearing saline test. In that individual, the maximum LV pressure generated with the saline test was 11 mmHg. There was only mild MR observed after separation from cardiopulmonary bypass, but a mild degree of residual posterior leaflet prolapse was observed. Additional neochordae were added in that patient with a postsurgical TEE confirming trace MR. These data maybe available upon reasonable request to the authors.

## COMMENT

Surgical mitral valve repair remains the gold standard approach for addressing MR. Since mitral valve reconstruction and manipulation is performed on the arrested heart, persistent MR may be observed following resumption of cardiac contraction. This scenario is unlike transcatheter edge-to-edge repair whereby valve repair is performed on the beating heart.

As an example, the saline test is an ubiquitous technique in performing surgical mitral valve repair. Unlike percutaneous mitral valve therapies, which are performed on the beating heart, the saline test provides the closest approximation to LV filling in the quiescent heart. Notwithstanding, the haemodynamics of the saline test have not been previously assessed. This series confirms that the saline test approximates a patients’ systemic diastolic pressure. As such, persistent MR may be observed at physiologic loading conditions. However, leaflet deployment does occur and the assessment of valve anatomy, whether scallop prolapse or restriction, can be performed with LV filling.

Although novel to cardiac surgery, intraprocedural haemodynamic measurements are often performed for patients undergoing transcatheter mitral valve repair. Indeed, the real-time nature of mitral valve repair has lent to insights on pressure gradients postrepair [8]. Beyond this, the impact of chamber compliance has also not been evaluated in surgical patients [9].

Notwithstanding, there are a variety of areas for exploration regarding the haemodynamics of surgical mitral valve patients. Intra-aortic pressure was not measured at the time of LV filling with the saline test. Although the Cosgrove basket retractor provides some occlusion of the aorta during the saline test, fluid may not be completely confined to the LV thereby influencing the pressure generated and measured. Concomitant injection of fluid into the aortic root to better ensure aortic valve closure was also not performed. As well, this series is small and does not provide concrete evidence for the use of LV pressure monitoring at the time of surgical mitral repair. There may also be unique pathologies, which preclude application of the saline test [10].

Undoubtedly, the LV pressure filling is influenced by non-modifiable patient factors such as LV size, geometry and compliance. However, the saline test remains an integral component of surgical mitral valve repair. In this limited series, all patients had favourable early results following mitral repair. However, 1 patient required a second cardiopulmonary bypass run to address mild MR, but with subtle residual posterior leaflet prolapse. In that patient, the maximum LV pressure generated with the saline test was only 11 mmHg despite the fact that the saline test was otherwise competent. Indeed, the visual appearance of the saline test for that patient did not suggest any residual prolapse and no saline was observed leaking back across the left atrium. It might be such that the LV compliance did not allow for higher pressures to be generated therefore a true assessment of the reconstructed mitral valve anatomy was limited. These data may be of help to a surgeon interpreting intraoperative saline tests with the hope of a competent mitral valve.

Overall, intraventricular pressure measurements have not been used to assess surgical mitral valve repair. Although assessed in this report in the context of the saline test for surgical repair of degenerative MR, pressure measurements may also be useful for repair of other mitral valve pathologies. As well, it may be useful in the contact of aortic valve repair/replacement for the assessment of intra-cavitary gradients.

## ETHICAL STATEMENT

This work was approved by our local human research ethics board.

**Conflict of interest:** none declared.

### Reviewer information

Interactive CardioVascular and Thoracic Surgery thanks Stefano Benussi, Amir Youssari, Taiju Watanabe and the other, anonymous reviewer(s) for their contribution to the peer review process of this article.
